# Antineutrophil cytoplasmic antibody-associated pachymeningitis: a systematic review of clinical features, diagnosis and treatment outcomes

**DOI:** 10.1007/s12026-026-09782-2

**Published:** 2026-05-01

**Authors:** Graziella Aguiar Santos Faria, Paula Baleeiro Rodrigues Silva, Gabriela Abrahão Allioni, Leandro Tavares Lucato, Tarso Adoni, Luiz Henrique Martins Castro, Guilherme Diogo Silva

**Affiliations:** 1 Neurology Division, Hospital das Clinicas HCFMUSP, Faculdade de Medicina, Universidade de Sao Paulo, Rua Doutor Enéas de Carvalho Aguiar, 255 – Cerqueira César, Sao Paulo, SP 05403-900 Brazil; 2 Neuroradiology Group, InRad, Hospital das Clinicas HCFMUSP, Faculdade de Medicina, Universidade de Sao Paulo, Sao Paulo, SP Brazil

**Keywords:** Antibodies, antineutrophil cytoplasmic, Autoimmune diseases, Vasculitis, Magnetic resonance imaging, Immunology, Systematic review

## Abstract

Antineutrophil cytoplasmic antibody (ANCA)-associated pachymeningitis is a rare inflammatory disorder of the dura mater that may occur in isolation or as part of systemic vasculitis. The clinical features and outcomes of proteinase 3 (PR3)- and myeloperoxidase (MPO)-ANCA-associated pachymeningitis remain poorly defined. We aimed to synthesize clinical presentation, investigations, and treatment outcomes to improve diagnosis and guide management. A systematic review was conducted in accordance with PRISMA guidelines. PubMed, Embase, and Scopus were searched to June 15, 2023. Eligible studies were case reports or series of patients with imaging-confirmed pachymeningitis and positive ANCA serology. Individual patient data were extracted, including demographics, clinical features, pathology, and outcomes. We identified 230 patients from 177 reports, including 108 MPO-ANCA–positive, 71 PR3-ANCA–positive, and 3 dual MPO/PR3-positive cases; 46 ANCA-positive cases had unspecified antigen specificity, and 2 were ELISA-negative but ANCA IIF-positive. Median age was 60 years; 53% were male. Headache and cranial neuropathies—particularly hearing loss, visual impairment, and diplopia—were predominant. Systemic involvement was present in 89% of cases, primarily affecting the ear, nose, throat, lungs, orbits and kidneys. Imaging often showed tentorium/falx, middle cranial fossa, and frontal convexities involvement. Inflammatory markers were raised in > 90%, and cerebrospinal fluid pleocytosis in 50%. Typical pathological features such as granulomas, vasculitis, or necrosis were documented in 66% of biopsied patients. At a median 9-month follow-up, mortality rate was 3.9% and relapse rate 23.5%. Rituximab (25%) was associated with lower rates of refractory disease. PR3- and MPO-ANCA-associated pachymeningitis typically presents with headache and cranial neuropathies, usually with systemic involvement. Relapse and incomplete recovery remain common despite treatment, underscoring the need for early recognition, targeted immunotherapy, and long-term follow-up.

**Supplementary Information:**

The online version contains supplementary material available at 10.1007/s12026-026-09782-2.

## Introduction

Pachymeningitis is an inflammatory condition of the dura mater which may occur as a manifestation of isolated or systemic infectious or autoimmune diseases [1–3]. Most cases of pachymeningitis remain idiopathic [1], highlighting the need for new biomarkers in the field.

Antineutrophil cytoplasmic antibodies (ANCA) can be identified in up to 30–50% of patients with pachymeningitis [1]. Patients who test positive for ANCA differ from those classified as idiopathic because of older age of onset, higher prevalence of otological symptoms, and higher frequency of elevated inflammatory markers [1].

ANCAs target antigens found in neutrophil granules and are typically identified using two main methodologies: indirect immunofluorescence (IIF) and antigen-specific immunoassays. IIF distinguishes two major patterns—cytoplasmic (c-ANCA) and perinuclear (p-ANCA)—while enzyme-linked immunosorbent assays (ELISA) detect specific antigens associated with ANCA-associated vasculitis (AAV): proteinase 3 (PR3-ANCA, most commonly associated with c-ANCA) and myeloperoxidase (MPO-ANCA, typically linked to p-ANCA) [5]. Given its higher accuracy, antigen-specific immunoassay is the preferred diagnostic approach when AAV is suspected [6].

In clinical practice, PR3-ANCA is most commonly associated with granulomatosis with polyangiitis (GPA), a condition characterized by necrotizing granulomatous inflammation and vasculitis that typically affects both the upper and lower respiratory tracts [7]. In contrast, MPO-ANCA is more frequently linked with microscopic polyangiitis (MPA), which presents with non-granulomatous vasculitis and predominantly affects the lungs and kidneys [4].

The dura mater contains a reservoir of myeloid cells that mediate immune surveillance of the central nervous system [8] and might also be targeted by ANCA. PR3- and MPO-ANCA-associated pachymeningitis frequently manifests alongside systemic and pathological features reported in other organs, but may also present as an isolated meningeal disease without typical histopathological findings in meningeal biopsy [9].

In the last decade, there has been an increasing number of publications about the potential of PR3-ANCA and MPO-ANCA to characterize a distinct subgroup of patients with pachymeningitis. However, our current knowledge is based on case reports and case series. This scenario creates an appropriate time for a systematic review to summarize clinical, imaging, and laboratory features of PR3- and MPO-ANCA-associated pachymeningitis.

We aimed to address the specific questions: (1) What are the most common neurological and systemic signs and symptoms of patients with pachymeningitis who test positive for PR3-ANCA or MPO-ANCA antibodies? (2) What are the most common investigation findings (neuroimaging, laboratory, and pathology) in PR3-ANCA and MPO-ANCA-associated pachymeningitis? and (3) What are the treatment outcomes of PR3-ANCA and MPO-ANCA-associated pachymeningitis?

## Methods

### Standard protocol approvals, registrations, and patient consents

This systematic review is reported in accordance with the Preferred Reporting Items for Systematic Reviews and Meta-Analyses (PRISMA) guidelines. The review protocol was registered in the International Prospective Register of Systematic Reviews (PROSPERO) under the identifier CRD42023425334. As this study used only published, de-identified data, institutional review board approval and patient consent were not required [10].

### Search strategy

We searched PubMed/MEDLINE, Embase, and Scopus from inception to June 15, 2023. The search strategy included terms for MPO, PR3, c-ANCA, p-ANCA, AAV subtypes, and pachymeningitis (Supplementary Data, Appendix 1). Reference lists of included studies were screened to identify additional relevant records.

### Selection criteria

The criteria for study inclusion were: (1) diagnosis of pachymeningitis based on cranial or spinal thickening with enhancement on magnetic resonance imaging (MRI), and (2) positive ANCA results, regardless of the specific immunological test used. Exclusion criteria were: (1) absence of individual patient data, (2) concern for dual reporting, and (3) evidence of another autoimmune disease that could manifest as pachymeningitis, such as elevated serum IgG4 levels. We considered overlap between ANCA and IgG4-related disease might create a specific phenotype of the disease and, hence, we excluded patients with elevated serum IgG4 levels. We did not exclude studies based on date or Language of publication.

### Data extraction and quality appraisal

Two independent reviewers (G.A.S.F. and P.B.R.), both neurologists with expertise in neuroimmunology, screened titles, abstracts, and full texts for eligibility. Disagreements were resolved through discussion with a third neuroimmunologist (G.D.S.). Duplicate records were identified using a semi-automated approach in Rayyan and confirmed through manual review. Studies were included if they reported individual-patient-level data from published case reports or case series.

Data extraction was performed independently by the same two reviewers using pilot-tested forms developed in the Research Electronic Data Capture (REDCap) platform, a secure web-based application for managing research data. Cases from the same research groups were cross-checked to avoid duplication.

Extracted data included demographic characteristics (sex, age, country), neurological symptoms, systemic involvement, imaging findings, ANCA testing methodology, laboratory and CSF parameters, histopathology, treatment details, and clinical/imaging outcomes. No formal risk-of-bias assessment was performed, given the inclusion of case reports and case series, which are inherently at high risk of bias by design.

### Outcomes

Treatment response was categorized as complete response, partial response, no response, or worsening at last follow-up, based on clinical and radiologic improvement. This classification has been previously applied in a systematic review of pachymeningitis in another condition, IgG4-related disease [2]. Refractory disease was defined as lack of significant clinical response or disease relapse despite treatment.

### Statistical analysis

Quantitative data were summarized using medians and interquartile ranges (IQR); qualitative data were expressed as frequencies and percentages. Comparative analyses between PR3- and MPO-ANCA cases were conducted using Chi-squared or Mann-Whitney U tests, as appropriate. In all analyses, denominators were adjusted according to data availability for each variable, with cases lacking relevant information excluded from specific calculations to appropriately account for missing data. A p-value < 0.05 was considered statistically significant. Analyses were performed using RStudio (2022.07.2).

### Data availability

All data analyzed during this study are included in the published article and its supplementary files. Further inquiries can be directed to the corresponding author.

## Results

We identified 1,535 records, of which 177 studies and 230 patients (115 MPO-ANCA+, 70 PR3-ANCA+, 45 ANCA+ unspecified) were included (Supplementary Data, eFigure 1 and eAppendix).

### Clinical features

Our study had 119/226 male patients (52.7%). The median age at presentation was 60 years (interquartile range [IQR]: 46–68). Most cases were reported from Japan (101; 43.9%), followed by the United States (28; 12.2%), China, Mexico (both 13; 5.6%), and the UK (10; 5.7%).

The neurological and systemic presentation of PR3- and MPO-ANCA-associated pachymeningitis is summarized in Table 1. Pachymeningitis manifested in a subacute manner, with a median duration of 3 months (IQR 1–7) prior to patients seeking medical attention. The most frequent presentation was headache, followed by cranial nerve dysfunction such as hearing loss, visual loss, and diplopia. The neurological presentation of PR3- and MPO-ANCA associated pachymeningitis was similar.

**Table 1:** Clinical features of PR3-ANCA and MPO-ANCA–associated pachymeningitis

Clinical features	ANCA (*n* = 229)	MPO-ANCA (*n* = 107)	PR3-ANCA (*n* = 71)
Neurological signs and symptoms			
Headache – n (%)	161 (70%)	70 (65%)	51 (72%)
Hearing loss – n (%)	64 (28%)	42 (39%)	18 (25%)
Visual loss – n (%)	57 (25%)	31 (29%)	19 (27%)
Diplopia – n (%)	46 (20%)	18 (17%)	16 (23%)
Facial palsy – n (%)	42 (18%)	25 (23%)	8 (11%)
Dysphagia – n (%)	19 (8%)	13 (12%)	4 (6%)
Seizures – n (%)	7 (3%)	2 (2%)	4 (6%)
Meningeal signs – n (%)	3 (1%)	1 (1%)	0 (0%)
Systemic involvement			
Any systemic involvement – n (%)*	203 (89%)	91 (85%)	70 (99%)
ENT – n (%)*	152 (66%)	68 (64%)	59 (83%)
Lung – n (%)**	69 (30%)	23 (22%)	33 (46%)
Orbital – n (%)*	63 (28%)	20 (19%)	28 (39%)
Kidney – n (%)	52 (23%)	24 (22%)	18 (25%)
Articular – n (%)*	31 (14%)	5 (5%)	12 (17%)
Cutaneous – n (%)*	14 (6%)	1 (1%)	7 (10%)

A prior diagnosis of AAV was present in only a third of patients. However, 89% of patients with ANCA-associated pachymeningitis had evidence of systemic disease. MPO-ANCA-associated pachymeningitis had a higher frequency of isolated meningeal disease (15%) compared with PR3-ANCA associated pachymeningitis. The most frequently involved organs were ear, nose, throat, lungs, orbits and kidneys.

### Investigation findings

Of the 230 patients included in the study, 229 were analyzed for topographic dural involvement. One article was excluded due to the lack of explicit mention of topographic involvement and the absence of imaging available for review (Table 2). The most frequently involved region was the tentorium / falx cerebelli.

**Table 2:** Imaging findings of PR3-ANCA and MPO-ANCA–associated pachymeningitis

Investigation findings	ANCA + (*n* = 230)	MPO + (*n* = 108)	PR3 + (*n* = 71)
Imaging findings			
Indeterminate, n (%)	50 (22%)	22 (20%)	17 (24%)
Skull base, n (%)	126 (55%)	64 (59%)	36 (51%)
Anterior fossa, n (%)	25 (11%)	15 (14%)	8 (11%)
Middle fossa, n (%)	51 (22%)	27 (25%)	14 (20%)
Posterior fossa, n (%)	4 (2%)	2 (2%)	0
Cavernous sinus, n (%)	23 (10%)	8 (7%)	9 (13%)
Orbital apex, n (%)	23 (10%)	12 (11%)	6 (8%)
Tentorium / falx cerebelli, n (%)	68 (30%)	37 (34%)	16 (23%)
Cerebellar convexities, n (%)	26 (11%)	13 (12%)	9 (13%)
Cerebellopontine angle, n (%)	15 (7%)	6 (6%)	6 (8%)
Clivus and retroclival space, n (%)	11 (5%)	4 (4%)	4 (4%)
Foramen magnum, n (%)	2 (1%)	1 (1%)	0
Cerebral convexities and falx cerebri, n (%)	101 (44%)	47 (44%)	37 (52%)
Frontal, n (%)	62 (27%)	24 (22%)	26 (3 × 7%)
Parietal, n (%) *	33 (14%)	9 (8%)	16 (23%)
Temporal, n (%)	45 (20%)	20 (19%)	18 (25%)
Occipital, n (%)	5 (2%)	3 (3%)	1 (1%)
Falx cerebri, n (%)	41 (18%)	21 (19%)	11 (15%)
Spinal involvement (*n* = 43), n (%)	21 (9%)	10 (9%)	2 (3%)
Cervical, n (%)	11 (52%)	7 (6%)	1 (1%)
Thoracic, n (%)	14 (67%)	6 (5%)	2 (3%)
Lumbar or sacral, n (%)	2 (10%)	1 (1%)	1 (1%)
Bilateral, n (%)	56 (25%)	29 (27%)	19 (27%)

Imaging findings are presented as number (percentage) of patients. Percentages were calculated within each column. Spinal involvement percentages for subregions are calculated relative to the total number of patients with spinal disease (*n* = 21). CSF: cerebrospinal fluid. * *p* < 0.05 for comparison between MPO-ANCA–positive and PR3-ANCA–positive groups

**Fig. 1:**
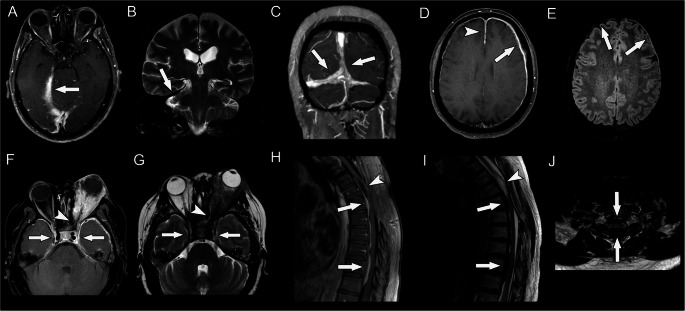
MR imaging findings in ANCA-associated pachymeningitis (patient 1, figures **A**-**C**; patient 2, figures **D**-**E**; patient 3, figures **F**-**G**; and patient 4, figures **H**-**J**). Axial postcontrast T1-weighted image (**A**) and coronal T2-weighted images (**B**) demonstrate dural thickening involving the right cerebellar tentorium, with contrast enhancement and T2-hypointensity (arrows). Coronal postcontrast T1-weighted image (**C**) shows extensive pachymeningeal involvement of tentorium and the posterior portion of the falx cerebri (arrows), resembling the so-called “Eiffel-by-Night” sign. Axial postcontrast T1-weighted **(D**) and FLAIR (**E**) images show pachymeningitis involving the frontoparietal convexity, more prominent to the right (arrows) and the anterior portion of the falx cerebri (arrowhead in D). Axial postcontrast T1-weighted image (**F**) and T2-weighted images (**G**) depict a large amount of a T2-hypointense tissue, with avid contrast enhancement, infiltrating the left orbit and causing proptosis Notice also the extension to the orbital apex (arrowheads) and both cavernous sinuses (arrows), together with dural thickening in the middle cranial fossa bilaterally, especially near the temporal poles. Sagittal postcontrast T1-weighted (**H**) and T2-weighted images (**I**) demonstrate dural thickening inside the thoracic vertebral canal with T2-hypointensity and contrast enhancement (arrows); there were also postoperative changes in the posterior portion of the upper thoracic spine together with myelopathy (arrowhead). Axial T2-weighted image of the same patient, in the cervicothoracic transition (**J**), shows spinal cord compression between anterior and posterior T2-hypointense pachymeningitis (arrows)

The skull base involvement predominated in middle fossa. One in ten patients had involvement of the cavernous sinus. The cerebral convexities involved presented an anteroposterior gradient, with a higher frequency of frontal and temporal pachymeningitis. Spinal pachymeningitis was most frequently located at cervical and thoracic segments. Figure 1 illustrates the spectrum of MRI findings observed in ANCA-positive pachymeningitis.

Concomitant leptomeningitis was rare, occurring in only 6 cases (2.6%). Parenchymal involvement was reported in 16 patients (7%), typically as a result of infiltration or mass effect (edema) adjacent to the meningeal disease. Additional findings included infarction in 10 cases (4.4%) and hemorrhage in 5 cases (2.2%). Thrombosis was noted in 13 patients (5.7%).

Table 3 summarizes the laboratory and pathology findings in patients with ANCA-positive pachymeningitis. Only half of the patients presented cerebrospinal fluid pleocytosis, all of which were lymphocytic in nature. CSF hypertension in 24 of 90 cases (26.7%).

**Table 3:** Laboratory and pathological findings of PR3-ANCA and MPO-ANCA–associated pachymeningitis

Investigation findings	ANCA + (*n* = 230)	MPO + (*n* = 108)	PR3 + (*n* = 71 )
CSF findings			
Cerebrospinal fluid			
- > 5 cells/mm^3^ – n (%)	48/100 (48%)	28/53 (53%)	8/24 (33%)
- Lymphocytic pleocytosis – n (%)	33/33 (100%)	20/20 (100%)	6/6 (100%)
- Elevated protein levels – n (%)*	63/102 (62%)	42/55 (76%)	10/24 (42%)
- Oligoclonal bands – n (%)	5/13 (38%)	2/6 (33%)	2/5 (40%)
- Elevated IgG levels – n (%)	14/21 (67)	12/15 (80%)	2/4 (50%)
Serum findings			
Elevated ESR – n (%)*	78/85 (92%)	37/37 (100%)	23/27 (85%)
Elevated CRP – n (%)	127/136 (93%)	73/79 (91%)	38/39 (97%)
Eosinophilia – n (%)	5/230 (2%)	4/108 (4%)	1/71 (1%)
Pathological findings			
Biopsy site			
- Meninges	47/101 (47%)	21/36 (58%)	11/31 (35%)
- Nasal sinus	32/101 (32%)	10/36 (27%)	12/31 (39%)
- Lung	13/101 (13%)	3/36 (8%)	7/31 (23%)
- Kidney	17/101 (17%)	8/36 (22%)	4/31 (13%)
- Other	19/101 (19%)	9/36 (25%)	4/31 (13%)
Necrotizing or non-necrotizing granuloma – n (%)	46/101 (46%)	13/36 (36%)	16/31 (52%)
Vasculitis – n (%)	44/101 (44%)	13/36 (36%)	17/31 (55%)
Necrosis – n (%)	40/101 (40%)	13/36 (36%)	14/31 (45%)

*CSF* cerebrospinal fluid, *ESR* erythrocyte sedimentation rate, *CRP* C-reactive protein.* *p* < 0.05 for comparison between MPO-ANCA–positive and PR3-ANCA–positive groups

We observed elevated ESR and CRP in more than 90% of patients who reported the results of these tests. MPO-ANCA or PR3-ANCA antibodies were reported in 180 (78%) patients, with MPO-ANCA being more frequently detected than PR3-ANCA. Thirteen patients tested negative by ELISA during the phase of pachymeningitis but were positive either before its onset or during follow-up; of these, 12 demonstrated positivity for MPO antibodies. Three patients (1.6%) were positive for both MPO and proteinase 3 (PR3) antibodies (see Table 4).

**Table 4:** Distribution of ANCA by IIF and ELISA Methods in Patients with ANCA-Associated Pachymeningitis

ANCA IIF and anti-MPO, anti-PR3 ELISA					
ANCA IIF (*n* = 105)	ELISA (*n* = 184)				
	MPO-ANCA (*n* = 108)	PR3-ANCA (*n* = 71)	Negative (*n* = 2)	Double (*n* = 3)	Not specified (*n* = 46)
p-ANCA (*n* = 44)	21	2	1	0	20
c-ANCA (*n* = 57)	3	30	1	0	23
ANCA-Negative (*n* = 3)	3	0	0	0	0
Double (*n* = 1)	0	0	0	0	1
Not specified (*n* = 125)	81	39	0	3	2*

Data are presented as absolute counts. ANCA results are stratified by IIF patterns (p-ANCA, c-ANCA, ANCA-negative, double, or not specified) and by ELISA specificity (MPO-ANCA, PR3-ANCA, negative, double, or not specified). Denominators vary according to data availability for each testing modality. *IIF* indirect immunofluorescence, *ELISA* enzyme-linked immunosorbent assay, *MPO* myeloperoxidase, *PR3* proteinase 3

*Cases reported as ANCA-associated pachymeningitis, but without specification of the detection method (IIF or ELISA)

In our study, pathological findings were documented in 101 patients, with 47% of these cases originating from meningeal biopsies. The three most common extra-meningeal sites for biopsy were nasal sinus, lung, and kidney. At least one of the specific pathological features—necrotizing granulomas, non-necrotizing granulomas, necrosis, or vasculitis—was identified in 68.3% of cases; however, the simultaneous presence of all these features was observed in less than half of patients. Granulomatous inflammation without vasculitis was seen in 21% of the ANCA-positive pachymeningitis biopsies, with necrotizing granulomas present in 10 of these 21 cases (see Table 3).

### Treatment outcomes

**Fig. 2:**
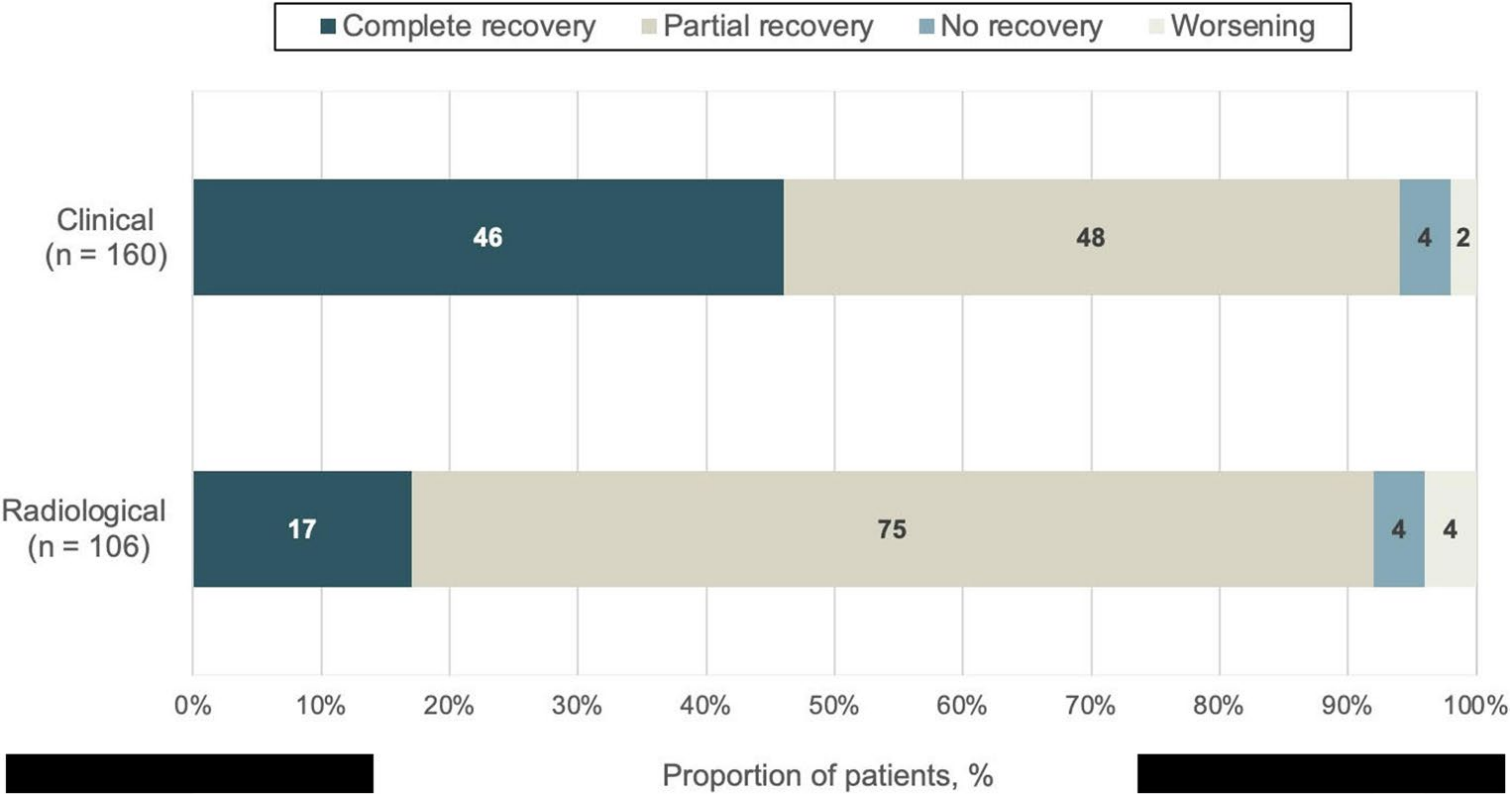
Clinical and radiological outcomes in ANCA-associated pachymeningitis

During a median follow-up of 9 months (IQR 3–24), nine patients (3.9%) died, and 56 (25.5%) experienced recurrences of pachymeningeal disease. The median time to first relapse of 7 months (IQR 3–12). Only three (5%) patients relapsed after four years, with the longest time to relapse being 72 months. Among those with relapses, 42 patients had one recurrence, four patients had two, three patients had three, and two patients experienced four relapses. Complete clinical recovery was achieved in 74 cases (46%), whereas complete radiological resolution was documented in 18 cases (17%) (Fig. 2).

Treatment data were reported for 207 studies. Glucocorticoids were the most frequently used therapy, prescribed for 206 patients (99.5%). Cyclophosphamide was the most commonly administered immunosuppressant, used in 108 patients (52.2%). Rituximab was prescribed as maintenance therapy in 50 (24.2%) patients. Among oral immunosuppressants, azathioprine (32, 15.5%) and methotrexate (31, 15%) were most common, followed by mycophenolate mofetil (9, 4.3%).

We compared outcomes based on the geographical distribution of publications. Case reports from Asia showed significantly higher rates of imaging improvement (97% versus 85%, *p* = 0.021) compared to reports from other regions. Relapse rates and follow-up durations were similar across all regions.

We also evaluated outcomes according to the pattern of dural involvement. Clinical and radiological outcomes were similar between patients with spinal and cranial involvement in ANCA-positive pachymeningitis. The use of different immunosuppressive agents was comparable between the two groups.

We assessed the frequency of refractory disease in patients treated with rituximab versus other immunosuppressive agents, with first-line rituximab used in a subset of patients (14.9%) and associated with no refractory disease compared with 56.6% among those receiving alternative first-line agents (*p* < 0.001).

## Discussion

This systematic review of 230 patients summarizes the available evidence on clinical presentation, investigation findings, and treatment outcomes of ANCA-associated pachymeningitis.

**Fig. 3:**
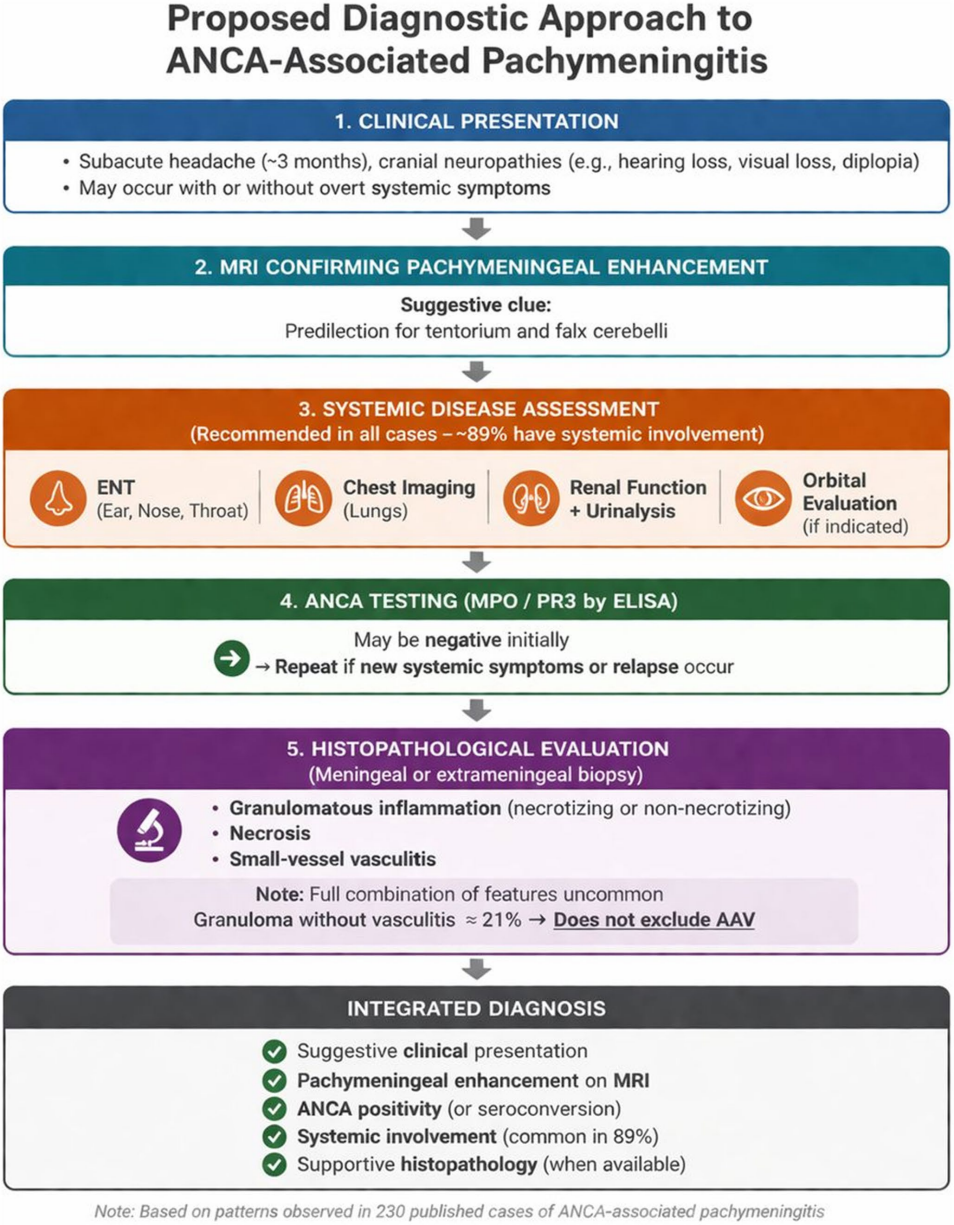
Proposed diagnostic approach to ANCA-associated pachymeningitis. A stepwise framework integrating clinical presentation, MRI findings, systematic evaluation for extracranial involvement, ANCA testing, and histopathological assessment. This approach is based on patterns observed across 230 published cases and is intended to support clinical decision-making rather than define prescriptive diagnostic criteria

Our findings support a structured diagnostic approach to ANCA-associated pachymeningitis integrating clinical features, MRI, evaluation for extracranial involvement, ANCA testing, and histopathology when feasible, as summarized in Fig. 3. This framework emphasizes early recognition and systematic assessment to support diagnosis. Patients with PR3-ANCA positivity, systemic vasculitis, and granulomatous inflammation may represent a phenotype more closely aligned with systemic ANCA-associated vasculitis and a higher-risk disease profile, whereas isolated MPO-ANCA pachymeningitis may reflect a more localized presentation, although careful longitudinal evaluation remains essential.

The most common neurological presentation of ANCA-associated pachymeningitis was headache, often accompanied with cranial nerve impairment. Hearing loss is observed more frequently in ANCA-associated pachymeningitis (28%) than in IgG4-related pachymeningitis (8%), as reported in a previous systematic review [2]. Isolated meningeal disease is less common in ANCA-associated pachymeningitis (11%) compared to IgG4-related pachymeningitis (51%)^2^, with a higher prevalence among MPO-ANCA patients (15%) than in PR3-ANCA cases (1%). Biopsies of the ENT region and lungs proved valuable in diagnosing both conditions. Caution is warranted due to the potential time lag between systemic manifestations and pachymeningeal involvement, as up to one-third of patients had a prior diagnosis of ANCA-associated vasculitis at the time of pachymeningitis; therefore, cases presenting as isolated disease may still evolve into systemic disease with longer follow-up.

The imaging presentation of ANCA-positive pachymeningitis has similarities with IgG4-related disease [2] such as: preferential involvement of middle fossa in skull base and preferential anterior involvement in supratentorial region. However, involvement of the tentorium and cerebellar falx is more frequent in ANCA-associated pachymeningitis (30%) than in IgG4-related pachymeningitis [2]. Future studies should evaluate whether the distribution pattern of pachymeningeal involvement can distinguish between these two conditions.

Granulomatous inflammation was observed in 46% of meningeal biopsies, occurring more frequently in PR3-ANCA cases (52%) compared to MPO-ANCA patients (35%). Importantly, granulomatous inflammation without vasculitis was found in 21% of ANCA-associated pachymeningitis biopsies, underscoring the potential for overlap with neurosarcoidosis, which typically features granulomas but lacks vasculitis [3]. Nearly half of these granulomatous cases exhibited necrotizing granulomas, demonstrating that granulomas in ANCA-associated pachymeningitis can be both necrotizing and non-necrotizing. Unlike sarcoidosis [3], ANCA-associated pachymeningitis rarely shows hilar lymphadenopathy and is distinguished by the presence of ANCA antibodies and pathological evidence of vasculitis and necrosis alongside granulomatous inflammation, highlighting the importance of careful clinicopathologic correlation to avoid misdiagnosis. We did not identify a consistent association between specific histopathological patterns and treatment response; although biopsy can be valuable for diagnosis, it may be challenging to obtain in resource-limited settings and did not appear to inform differential treatment response in our study.

This distinction is clinically significant, as ANCA-associated pachymeningitis has been observed to respond particularly well to rituximab. One possible explanation for this therapeutic response is the central role of B lymphocytes in ANCA-associated vasculitis; activated B cells contribute to disease activity and autoantibody production, and their depletion via rituximab may effectively disrupt this pathogenic process [11]. In contrast, sarcoidosis-related pachymeningitis has shown greater responsiveness to anti-TNF-alpha biologics in refractory cases 3. This underscores the importance of accurate differential diagnosis in guiding targeted immunotherapy.

The distinction between induction and maintenance regimens, as well as treatment duration, was inconsistently reported, precluding a systematic evaluation of optimal therapeutic sequencing and duration; to mitigate this limitation, we grouped patients according to rituximab versus other immunosuppressive therapies to provide insight into the use of this biologic agent, for which there is evidence of benefit in other organ manifestations. Rituximab was used in a subset of patients and was associated with favourable outcomes in reported cases; however this observation is limited by potential publication and selection biases. As rituximab is typically reserved for patients with more severe or refractory disease, a higher baseline risk of unfavorable outcomes would be expected in this group. However, no refractory cases were observed among patients receiving rituximab as initial therapy; suggesting a lower impact of this bias in our results.

The 25% relapse rate and the median time to first relapse of 7 months following a severe manifestation of ANCA-associated disease highlight the need for maintenance immunosuppression. The optimal duration of maintenance therapy remains a matter of debate, but it is often considered longer in severe manifestations, such as pachymeningitis, typically ranging from 18 months to 4 years. As 95% of relapses occurred within the first four years, it is reasonable to maintain therapy during this period, particularly when rituximab is used, given its combination of efficacy and a favorable safety profile.

Among patients with ANCA-associated pachymeningitis, myeloperoxidase was the most commonly identified antigen. We observed cases with isolated pachymeningitis and MPO-ANCA positivity, suggesting that pachymeningitis may represent an organ-specific form of vasculitis confined to the meninges. However, other cases showed PR3 positivity or an unspecified antigen, which might indicate a reaction to additional antigens in ANCA-positive patients. Future studies should aim to clarify whether ANCA positivity in pachymeningitis is associated with specific antigens that require further characterization.

This study has some limitations. The median follow-up period of only 9 months may underestimate the true relapse rate of ANCA-associated pachymeningitis; however, even within this brief period, the relapse frequency was significant. By restricting inclusion to ANCA-positive cases, our study may not fully capture the spectrum of AAV-related pachymeningitis, particularly ANCA-negative presentations such as GPA; however, this approach allowed for a more homogeneous cohort and a more specific characterization of features associated with ANCA seropositivity. Data on disease activity scores were not consistently reported, precluding their systematic analysis. Moreover, we focused this study on pachymeningitis and its complications, and peripheral nervous system involvement was not systematically assessed; therefore, it could not be analyzed. Additionally, publication bias might have influenced the reported ANCA antigen positivity, as cases with ANCA positivity in the absence of MPO-ANCA or PR3-ANCA might have been dismissed as false positives and thus not published as ANCA-associated pachymeningitis. Despite these limitations, this review provides data from a substantial number of patients with this rare disease, offering valuable insights for clinicians.

In conclusion, headache and cranial neuropathies were the most common neurological manifestations of ANCA-associated pachymeningitis. MPO-ANCA was the predominant antibody identified, and although rare, isolated meningeal disease was observed. Imaging most frequently revealed involvement of the tentorium and middle cranial fossa, and histopathology demonstrated granulomatous inflammation with necrosis and vasculitis in nearly half of the cases. Rituximab as first-line therapy was associated with a lower frequency of refractory disease compared to other immunosuppressants. Altogether, these findings support the recognition of ANCA-associated pachymeningitis as a distinct and clinically relevant cause of pachymeningitis.

## Supplementary Information

Below is the link to the electronic supplementary material.

Supplementary Material 1 (DOCX 88.0 KB)

## Data Availability

All data analyzed during this study are included in the published article and its supplementary files. Further inquiries can be directed to the corresponding author.

## References

[1] Yonekawa T, Murai H, Utsuki S, et al. A nationwide survey of hypertrophic pachymeningitis in Japan. J Neurol Neurosurg Psychiatry. 2014;85(7):732–9. 24273222 10.1136/jnnp-2013-306410

[2] Terrim S, Mahler JV, Filho FVM, et al. Clinical presentation, investigation findings, and outcomes of IgG4-related pachymeningitis: a systematic review. JAMA Neurol Published online November. 2024;18. 10.1001/jamaneurol.2024.3947. 10.1001/jamaneurol.2024.3947 39556369

[3] Chakales P, Herman M, Chien LC, Hutto S. Pachymeningitis in biopsy-proven sarcoidosis: clinical course, radiographic findings, response to treatment, and long-term outcomes. Neurology. 2022;99(23):S63–4. 10.1212/NXI.0000000000200028 PMC9513981 36163175

[4] Arnold S, Kitching AR, Witko-Sarsat V, et al. Myeloperoxidase-specific antineutrophil cytoplasmic antibody-associated vasculitis. Lancet Rheumatol. 2024;6(5):e300–13. 38574743 10.1016/S2665-9913(24)00025-0

[5] Damoiseaux J. (2021). ANCA testing in clinical practice: from implementation to quality control and harmonization. Front Immunol. 2021;12:656796. 10.3389/fimmu.2021.656796 PMC8008144 33796118

[6] Bossuyt X, Tervaert C, Arimura JW. Position paper: Revised 2017 international consensus on testing of ANCAs in granulomatosis with polyangiitis and microscopic polyangiitis. Nat Rev Rheumatol. 2017;13(11):683–92. 28905856 10.1038/nrrheum.2017.140

[7] Falde SD, Fussner LA, Tazelaar HD, et al. Proteinase 3-specific antineutrophil cytoplasmic antibody-associated vasculitis. Lancet Rheumatol. 2024;6(5):e314–27. 38574742 10.1016/S2665-9913(24)00035-3

[8] Cugurra A, Mamuladze T, Rustenhoven J, et al. Skull and vertebral bone marrow are myeloid cell reservoirs for the meninges and CNS parenchyma. Science. 2021;373(6553):eabf7844. 34083447 10.1126/science.abf7844 PMC8863069

[9] Shimojima Y, Sekijima Y. Hypertrophic pachymeningitis in ANCA-associated vasculitis: Clinical and immunopathological features and insights. Autoimmun Rev. 2023;22(6):103338. 37062439 10.1016/j.autrev.2023.103338

[10] Page MJ, McKenzie JE, Bossuyt PM, et al. The PRISMA 2020 statement: an updated guideline for reporting systematic reviews. BMJ. 2021;372:n71. 33782057 10.1136/bmj.n71 PMC8005924

[11] Keogh KA, Ytterberg SR, Fervenza FC, Carlson KA, Schroeder DR, Specks U. Rituximab for refractory Wegener’s granulomatosis: report of a prospective, open-label pilot trial. Am J Respir Crit Care Med. 2006;173:180–7. 16224107 10.1164/rccm.200507-1144OC PMC2662987

