# Supplementary material for: Antineutrophil cytoplasmic antibody-associated pachymeningitis: a systematic review of clinical features, diagnosis and treatment outcomes
Source: Immunol Res. 2026 May 1;74(1):47. doi: 10.1007/s12026-026-09782-2 (PMC13132928; doi:10.1007/s12026-026-09782-2)
Supplement: Supplementary file 1 — Supplementary Material 1 (DOCX 88.0 KB) [file 12026_2026_9782_MOESM1_ESM.docx]

**Supplementary Online Content**

**Antineutrophil Cytoplasmic Antibody-Associated Pachymeningitis: A Systematic Review of Clinical Features, Diagnosis and Treatment Outcomes**

**eAppendix**. List of case reports ----------------------------------------------------------------------------------------------p2

**eFigure 1**. Flow diagram ------------------------------------------------------------------------------------------------------p16

**eAppendix**. List of case reports

1. Seino H, Nagahata F, Nagahata M, et al. A case of idiopathic hypertrophic spinal pachymeningitis: interesting dural signal intensity on repeat MR Imaging. *Neuroradiology*. 2010;52(7):678-679. doi:10.1007/s00234-010-0721-6
2. Rong Y, Jhala N. Cranial Hypertrophic Pachymeningitis and Granulomatosis with Polyangiitis: A Case Report and Review of the Literature. *J Neuropathol Exp Neurol*. 2021;80(6):577. doi:10.1093/jnen/nlab042
3. Freifeld AJ, Mutter M. Delayed diagnosis of vasculitis in a patient with chronic mastoiditis. *J Gen Intern Med*. 2021;36(0):S220. doi:10.1007/s11606-021-06830-5
4. Batista Blasco JL, Ciano Petersen NL, Cabezudo García P, Rodríguez Lavado I, Pons Pons G, Serrano Castro P. Long‐term risk of stroke and its predictors in transient ischaemic attack patients in Germany. *Eur J Neurol*. 2020;27(4):723-724. doi:10.1111/ene.14136
5. Los O, Asif A, Delpirou Nouh C, Al-Awwad A, Bhrugav R. Horner’s syndrome with multiple cranial nerve palsies: An unusual neurological manifestation of granulomatosis with polyangiitis. *Ann Neurol*. 2021;90(S27):S125. doi:10.1002/ana.26180
6. Xydakis I, Papageorgiou E, Psychoyios K, Koukouni V, Gkekas G. Hypertrophic pachymeningitis as a neurological manifestation of p-ANCA angiitis. *J Neurol*. 2012;259(S1):S141. doi:10.1007/s00415-012-6524-4
7. Cárdenas H, Prieto N, Rubio M. Hypertrophic pachymeningitis as an unusual presentation of central nervous system limited vasculitis. *Journal of Clinical Rheumatology*. 2020;20(3):S136-S137.
8. Syed A, Masroujeh R, Teba C. MPO-ANCA associated hypertrophic pachymeningitis. *Am J Respir Crit Care Med*. 2019;199(9).
9. Vandergheynst F, Bachir H. Pachymeningitis associated with ANCA-related vasculitis: About a refractory case. *Acta Clin Belg*. 2012;67(6):452.
10. Flores-Suárez LF, Higuera V, Ruiz N. (PM) in granulomatosis with polyangiitis (GPA). *Nephron*. 2015;129(2):153. doi:10.1159/000381120
11. Ledinek AH, Stokin CLS, Vesnaver T V. P-ANCA isolated cranial pachymeningitis: A case report. *J Neurol*. 2014;261(S1):S454. doi:10.1007/s00415-014-7337-4
12. Zeglam S, Werthmann M, Scheicht D, Bansmann P, Strunk J. Successful treatment of ANCA-associated vasculitis and CNS involvement with rituximab. *Z Rheumatol*. 2013;72(S2):17-163. doi:10.1007/s00393-013-1255-1
13. Ali T, Clements-Baker M. Would-be mimickers of vasculitis. *J Rheumatol*. 2018;45(7):1060. doi:10.3899/jrheum.180300
14. Wingerchuk DM, Dyck PJ, Mertz LE. Hemi-meningitis: A focal sign heralding a multisystem necrotizing vasculitis. *Neurology.* 2008;70(21):2014. doi:10.1212/01.wnl.0000312520.71743.54.
15. Rogister F, Kaye O, Tebache M, Daele J. Quand la rhinosinusite révèle une pathologie systémique [When rhinosinusitis reveals a systemic disease]. *Rev Med Liege.* 2015;70(2):78-85.
16. Levin A, Kasem S, Mader R, Naparstek Y, Friedman G, Ben-Yehuda A. Wegener granulomatosis with back pain, periaortitis, and dural inflammation developing while receiving monthly cyclophosphamide. *J Clin Rheumatol.*2006;12(6):294-297. doi:10.1097/01.rhu.0000249863.76020.dd.
17. Tamura N, Matsudaira R, Hirashima M, Ikeda M, Tajima M, Nawata M, Morimoto S, Kaneda K, Kobayashi S, Hashimoto H, Takasaki Y. Two cases of refractory Wegener's granulomatosis successfully treated with rituximab. *Intern Med.* 2007;46(7):409-414. doi:10.2169/internalmedicine.46.6156. PMID:17409608.
18. Bachmeyer C, Cervera P, Marro B, Ammouri W, Bouvard E, Favrole P, Jacquot F, Grateau G. Thoracic spinal cord compression indicating Wegener's granulomatosis in a patient with a previous presumptive diagnosis of microscopic polyangiitis. *Joint Bone Spine.* 2007;74(4):382-384. doi:10.1016/j.jbspin.2006.10.008. Erratum in: *Joint Bone Spine.* 2008;75(4):515. PMID:17587626.
19. Culbertson CJ, Lummus SC, Gold CA. Teaching NeuroImages: Myeloperoxidase-anti-neutrophil cytoplasmic antibody-positive hypertrophic pachymeningitis. *Neurology.* 2017;89(21):e253. doi:10.1212/WNL.0000000000004673. PMID:29158303.
20. Matsudaira R, Tamura N, Nawata M, Kaneda K, Takasaki Y. Successful treatment with rituximab in a refractory Wegener's granulomatosis with hypertrophic pachymeningitis and the right orbital granuloma. *Nihon Naika Gakkai Zasshi.* 2007;96(7):1464-1466. doi:10.2169/naika.96.1464. PMID:17682433.
21. Hermann J, Reittner P, Scarpatetti M, Graninger W. Successful treatment of meningeal involvement in Wegener's granulomatosis with infliximab. *Ann Rheum Dis.* 2006;65(5):691-692. doi:10.1136/ard.2005.043885. PMID:16611871; PMCID:PMC1798150.
22. Sharma A, Kumar S, Wanchu A, Lal V, Singh R, Gupta V, Singh S, Gupta A. Successful treatment of hypertrophic pachymeningitis in refractory Wegener's granulomatosis with rituximab. *Clin Rheumatol.* 2010;29(1):107-110. doi:10.1007/s10067-009-1291-z. Epub 2009 Oct 3. PMID:19802640.
23. Endo Y, Koga T, Ishida M, Fujita Y, Tsuji S, Takatani A, Shimizu T, Sumiyoshi R, Igawa T, Umeda M, Fukui S, Nishino A, Kawashiri SY, Iwamoto N, Ichinose K, Tamai M, Nakamura H, Origuchi T, Kawakami A. Rituximab-induced acute thrombocytopenia in granulomatosis with polyangiitis. *Intern Med.* 2018;57(15):2247-2250. doi:10.2169/internalmedicine.0335-17. Epub 2018 Mar 9. PMID:29526948; PMCID:PMC6120828.
24. Lim EJ, Kim SH, Lee SH, Lee KY, Choi JH, Nam EJ, Lee SH. Reversible sensorineural hearing loss due to pachymeningitis associated with elevated serum MPO-ANCA. *Clin Exp Otorhinolaryngol.* 2011;4(3):155-158. doi:10.3342/ceo.2011.4.3.155. Epub 2011 Feb 7. PMID:21949583; PMCID:PMC3173708.
25. Hasegawa H, Kohsaka H, Takada K, Miyasaka N. Renal involvement in antimyeloperoxidase antineutrophil cytoplasmic antibody-positive granulomatosis with polyangiitis with chronic hypertrophic pachymeningitis. *The Journal of Rheumatology.* 2012;39(10):2053-2055. doi:10.3899/jrheum.120473.
26. Bawa S, Mukhtyar C, Edmonds S, Webley M. Refractory Wegener's meningitis treated with rituximab. *J Rheumatol.* 2007;34(4):900-901. PMID:17407257.
27. Kimura Y, Asako K, Kikuchi H, Kono H. Refractory optic perineuritis due to granulomatosis with polyangiitis successfully treated with methotrexate and mycophenolate mofetil combination therapy. *Eur J Rheumatol.*2017;4(1):70-72. doi:10.5152/eurjrheum.2016.028. Epub 2017 Mar 1. PMID:28293459; PMCID:PMC5335893.
28. Horai Y, Miyamura T, Takahama S, Hirata A, Nakamura M, Ando H, Minami R, Yamamoto M, Suematsu E. Refractory antineutrophil cytoplasmic antibody-associated vasculitis successfully treated with rituximab: a case report. *Nihon Rinsho Meneki Gakkai Kaishi.* 2010;33(2):105-110. doi:10.2177/jsci.33.105. PMID:20453447.
29. Psenak O, Greil R. Response to rituximab after failure of cyclophosphamide in the induction treatment in a patient with cANCA-associated vasculitis and pachymeningitis: a case report. *Modern Rheumatology Journal.*2021;15(4):68-71. doi:10.14412/1996-7012-2021-4-68-71.
30. Higuera-Ortiz V, Reynoso A, Ruiz N, Delgado-Hernández RD, Gómez-Garza G, Flores-Suárez LF. Pachymeningitis in granulomatosis with polyangiitis: case series with earlier onset in younger patients and literature review. *Clin Rheumatol.* 2017;36(4):919-924. doi:10.1007/s10067-016-3520-6. Epub 2016 Dec 23. PMID:28012056.
31. Sakellariou GT, Kefala N. Pachymeningitis in granulomatosis with polyangiitis: a case report and a review of the literature. *Case Rep Rheumatol.* 2013;2013:840984. doi:10.1155/2013/840984.
32. Nagashima T, Maguchi S, Terayama Y, Horimoto M, Nemoto M, Nunomura M, Mori M, Seki T, Matsukawa S, Itoh T, Nagashima K. P-ANCA-positive Wegener's granulomatosis presenting with hypertrophic pachymeningitis and multiple cranial neuropathies: case report and review of literature. *Neuropathology.* 2000;20(1):23-30. doi:10.1046/j.1440-1789.2000.00282.x. PMID:10935433.
33. Salvi F, Mascalchi M, Pasini E, Bartolomei I, Fini N, Marliani F, Malatesta R, Michelucci R. p-ANCA pachymeningitis presenting with isolated "optic neuropathy". *Neurol Sci.* 2010;31(5):639-41. doi:10.1007/s10072-010-0283-8. Epub 2010 May 18. PMID:20480198.
34. Nakajima H, Yamane K, Kimura F, Oku H. Optic perineuritis associated with antineutrophil cytoplasmic autoantibody-related hypertrophic pachymeningitis: a case report. *Neurol Sci.* 2016;37(4):641-643. doi:10.1007/s10072-015-2454-0. Epub 2015 Dec 22. PMID:26694175.
35. Newman NJ, Slamovits TL, Friedland S, Wilson WB. Neuro-ophthalmic manifestations of meningocerebral inflammation from the limited form of Wegener's granulomatosis. *Am J Ophthalmol.* 1995;120(5):613-621. doi:10.1016/s0002-9394(14)72208-1. PMID:7485363.
36. Iqbal A, Blackburn D, Rafiq M, et al. Multiple cranial nerve palsies in a young singer. *J Neurol Neurosurg Psychiatry.*2012;83:A18.
37. Akahoshi M, Yoshimoto G, Nakashima H, Miyake K, Inoue Y, Tanaka Y, Tsukamoto H, Horiuchi T, Otsuka T, Harada M. MPO-ANCA-positive Wegener's granulomatosis presenting with hypertrophic cranial pachymeningitis: case report and review of the literature. *Mod Rheumatol.* 2004;14(2):179-183. doi:10.1007/s10165-004-0288-3. PMID:17143671.
38. Funayama Y, Watanabe G, Tsukita K, Suzuki H, Uenohara H, Suzuki Y. MPO-ANCA-associated hypertrophic pachymeningitis with monoclonal gammopathy of undetermined significance: a case report. *Rinsho Shinkeigaku.*2020;60(7):500-503. doi:10.5692/clinicalneurol.60.cn-001411. Epub 2020 Jun 13. PMID:32536662.
39. Horino T, Ichii O, Terada Y. Antineutrophil cytoplasmic antibody-associated hypertrophic pachymeningitis mimicking petroclival meningioma. *JCR: J Clin Rheumatol.* 2020;26(6):e153-e154. doi:10.1097/RHU.0000000000000958.
40. Clément M, Néel A, Toulgoat F, Weber M, Godmer P, Hutin P, Hamidou M, Lebranchu P. Inflammatory optic neuropathy in granulomatosis with polyangiitis can mimick isolated idiopathic optic neuritis. *Eur J Ophthalmol.* 2021;31(1):245-251. doi:10.1177/1120672119889008. Epub 2019 Nov 19. PMID:31744325.
41. Takagi T, Okada M, Nakamura M, Hanari T, Nakata T, Teraoka M, Hato N. Imaging characteristics of hypertrophic pachymeningitis due to ANCA-associated vasculitis. *Auris Nasus Larynx.* 2022;49(5):875-879. doi:10.1016/j.anl.2022.01.009. Epub 2022 Jan 21. PMID:35074241.
42. Zhao M, Geng T, Qiao L, Shi J, Xie J, Huang F, Lin X, Wang J, Zuo H. Idiopathic hypertrophic pachymeningitis: clinical, laboratory and neuroradiologic features in China. *J Clin Neurosci.* 2014;21(7):1127-1132. doi:10.1016/j.jocn.2013.09.025. Epub 2013 Dec 13. PMID:24589555.
43. Wu M, Ren J, Luo Y. Hypertrophic spinal pachymeningitis caused by ANCA-associated vasculitis revealed by 18F-FDG PET/CT: A case report. *Medicine (Baltimore).* 2021;100(3):e24388. doi:10.1097/MD.0000000000024388. PMID:33546079; PMCID:PMC7837913.
44. Mazzocchi O, Risso JA, Viozzi F, Apa NP, Peralba MC, Nadal MA, De Rosa GE. Paquimeningitis hipertrofica, glomerulonefritis y vasculitis de pequeños vasos asociada a ANCA [Hypertrophic pachymeningitis, glomerulonephritis and P-ANCA associated small vessel vasculitis]. *Medicina (B Aires).* 2007;67(2):151-5. PMID:17593600.
45. Shimojima Y, Kishida D, Hineno A, Yazaki M, Sekijima Y, Ikeda S. Hypertrophic pachymeningitis in a population with anti-neutrophil cytoplasmic antibody-associated vasculitis: a retrospective study in a single Japanese institution. Presented at: 2016 ACR/ARHP Annual Meeting; 2016 Sep 28; Washington, DC. Abstract 1935.
46. Furukawa Y, Matsumoto Y, Yamada M. Hypertrophic pachymeningitis as an initial and cardinal manifestation of microscopic polyangiitis. *Neurology*. 2004 Nov 9;63(9):1722-1724. doi: 10.1212/01.WNL.0000143063.12569.FC.
47. Yasutake M, Yukawa N, Iwahashi Y, et al. Hypertrophic pachymeningitis and subdural haematoma in an otitis media with ANCA-associated vasculitis patient successfully treated with intravenous cyclophosphamide. *Modern Rheumatology Case Reports*. 2019 Jul 3;3(2):134-138. doi: 10.1080/24725625.2019.1595945.
48. Di Stefano V, Dono F, De Angelis MV, Onofrj M. Hypertrophic pachymeningitis and cerebral venous thrombosis in myeloperoxidase-ANCA associated vasculitis. *BMJ Case Rep*. 2019 Jan 10;12(1):bcr-2018-226780. doi: 10.1136/bcr-2018-226780. PMID: 30635307; PMCID: PMC6340541.
49. Pensato U, Benini M, Fabbri VP, Avoni P, Foschini MP, Rizzo G, Liguori R. Headache and dural enhancement: two case studies of different treatable pathologies. *World Neurosurg*. 2020 Sep;141:306-310. doi: 10.1016/j.wneu.2020.06.126. Epub 2020 Jun 25. PMID: 32593763.
50. Ludueña A, Dorado E, Sarano J, Semeniuk G. Meningitis granulomatosa, glomerulonefritis rapidamente progresiva y vasculitis. *Medicina (B Aires)*. 2011;71(4):369-372. PMID: 21893452.
51. Kramer J, Frey J, Murray A. Granulomatosis with Polyangiitis-Associated Hypertrophic Pachymeningitis as a Neurosarcoidosis Mimic. *Autoimmune Neurology*. 2020;94(15_supplement):1821. doi:10.1212/WNL.94.15_supplement.1821.
52. Kato E, Tahara K, Hayashi H, Shoji A, Mori H, Sawada T. Granulomatosis with polyangiitis complicated by hypertrophic pachymeningitis presenting with simultaneous multiple intracerebral hemorrhages. *Intern Med*. 2018;57(8):1167-1172. doi:10.2169/internalmedicine.9660-17. Epub 2017 Dec 27. PMID: 29279498; PMCID: PMC5938513.
53. Benucci M, Li Gobbi F, Panconesi P, Manfredi M, Sarzi-Puttini P, Atzeni F. Granulomatosis polyangiitis associated with meningeal involvement: response to rituximab therapy after failure of cyclophosphamide. *Reumatismo*. 2013;65(2):90-94. doi:10.4081/reumatismo.2013.90. PMID: 23877415.
54. Sailler LJ, Porte L, Ollier SM, Astudillo LM, Couret BG, Catalaa I, Le Guellec S, Uro-Coste EV, Massip P, Arlet PM. Giant cell arteritis and spinal cord compression: an overlap syndrome? *Mayo Clin Proc*. 2006;81(1):89-91. doi:10.4065/81.1.89. PMID: 16438484.
55. Hayashi Y, Sugawara H, Otsuka M, Yamada S, Tabei K, Ueki A. Fatal hemoperitoneum preceded by cranial hypertrophic pachymeningitis in a patient with ANCA-positive microscopic polyangitis. *Intern Med*. 2008;47(11):1061-3. doi:10.2169/internalmedicine.47.0505. Epub 2008 Jun 2. PMID: 18520122.
56. Durant C, Martin J, Godmer P, Moreau A, Masseau A, Hamidou M. Exceptional osseous and meningeal spinal localization of ANCA-associated granulomatous vasculitis with hypertrophic spinal pachymeningitis. *J Neurol*. 2011 Jun;258(6):1172-3. doi:10.1007/s00415-010-5886-8. Epub 2011 Jan 6. PMID: 21210138.
57. Kobayashi K, Nakagomi D, Furuta S, Kobayashi Y, Hanai S, Yamagata M, Kawashima H, Kasuya T, Furuya H, Hiraguri M, Sugiyama T, Nakajima H. Efficacy of rituximab for anti-neutrophil cytoplasmic antibody-associated hypertrophic pachymeningitis: a case series. *Clin Exp Rheumatol*. 2020 Mar-Apr;38 Suppl 124(2):176-181. Epub 2020 May 21. PMID: 32441642.
58. Liewluck T, Schatz NJ, Potter PF, Romaguera RL. Compressive retrobulbar optic neuropathy due to hypertrophic pachymeningitis. *Intern Med*. 2008;47(19):1761-2. doi: 10.2169/internalmedicine.47.1412. Epub 2008 Oct 1. PMID: 18827435.
59. Kuribayashi T, Manabe Y, Fujiwara S, Omote Y, Narai H, Abe K. Combined hypertrophic pachymeningitis and cerebral venous thrombosis in a case of granulomatosis with polyangiitis. *Case Rep Neurol*. 2019 Aug 21;11(2):252-255. doi: 10.1159/000502284. PMID: 31572162; PMCID: PMC6751471.
60. Cañas CA, Díaz-Martínez JC, Tobón GJ. Combination of hypertrophic pachymeningitis, PR3-ANCA-positive vasculitis, and relapsing polychondritis. *J Rheumatol*. 2011 May;38(5):966-7. doi: 10.3899/jrheum.101238. PMID: 21532066.
61. Frey J, Kramer J, Castellani R, Sriwastava S. Clinical Reasoning: A 47-Year-Old With Headache, Vertigo, and Double Vision. *Neurology*. 2021 Aug 3;97(5):e535-e539. doi: 10.1212/WNL.0000000000012138.
62. Martin-Nares E, Cano Cruz LG, Hinojosa-Azaola A. Clinical Characteristics and Outcomes of ANCA-Associated and Non Immune-Mediated Hypertrophic Pachymeningitis: A Comparative Study. Abstract presented at: ACR Convergence; 2021; Virtual Meeting.
63. Zhao P, Zhang BS. Clinical analyses of perinuclear antineutrophil cytoplasmic antibody associated hypertrophic pachymeningitis. *Zhonghua Yi Xue Za Zhi*. 2013;93(11):837-40. PMID: 23859390.
64. Mekinian A, Maisonobe L, Boukari L, Melenotte C, Terrier B, Ayrignac X, Schleinitz N, Sène D, Hamidou M, Konaté A, Guilpain P, Abisror N, Ghrenassia E, Lachenal F, Cevallos R, Roos-Weil R, Du LTH, Lhote F, Larroche C, Bergmann JF, Humbert S, Fraison JB, Piette JC, Guillevin L, Dhote R, Amoura Z, Haroche J, Fain O. Characteristics, outcome and treatments with cranial pachymeningitis: A multicenter French retrospective study of 60 patients. *Medicine (Baltimore)*. 2018;97(30):e11413. doi: 10.1097/MD.0000000000011413. Erratum in: *Medicine (Baltimore)*. 2018;97(33):e12063. doi: 10.1097/MD.0000000000012063. PMID: 30045263; PMCID: PMC6078725.
65. Seror R, Mahr A, Ramanoelina J, Pagnoux C, Cohen P, Guillevin L. Central nervous system involvement in Wegener granulomatosis. *Medicine (Baltimore)*. 2006;85(1):53-65. doi: 10.1097/01.md.0000200166.90373.41. PMID: 16523054.
66. Nakamura T, Hirakawa K, Higashi S, Tomoda K, Tsukano M, Iyama K, Sakae T. CD8+ T lymphocytes infiltrate predominantly in the inflammatory foci of MPO-ANCA-positive thoracic hypertrophic pachymeningitis in a patient with HLA-A24. *Mod Rheumatol*. 2007;17(1):75-80. doi: 10.1007/s10165-006-0537-8. Epub 2007 Feb 20. PMID: 17278028.
67. Castrodad-Molina R, Borrero-Quintana E, Garcia Ayala ME, Blas-Boria D. Case of hypertrophic pachymeningitis with positive MPO-ANCA antibodies (P1.2-025). *Neurology*. 2019;92(15_supplement):P1.2-025. doi: 10.1212/WNL.92.15_supplement.P1.2-025.
68. Mori A, Hira K, Hatano T, Okuma Y, Kubo S, Hirano K, Ohno K, Noda K, Suzuki H, Ohsawa I, Hattori N. Bilateral facial nerve palsy due to otitis media associated with myeloperoxidase-antineutrophil cytoplasmic antibody. *Am J Med Sci*. 2013 Sep;346(3):240-3. doi: 10.1097/MAJ.0b013e318288371f. PMID: 23470272.
69. Parperis K, Abdulqader Y. Aortitis and pachymeningitis: an unusual combination in granulomatosis with polyangiitis (myeloperoxidase-associated vasculitis). *BMJ Case Rep*. 2019 Jan 28;12(1):e226795. doi: 10.1136/bcr-2018-226795. PMID: 30696638; PMCID: PMC6350718.
70. Kaga H, Komatsuda A, Saito M, Nara M, Omokawa A, Togashi M, Okuyama S, Wakui H, Takahashi N. Anti-neutrophil cytoplasmic antibody-associated vasculitis complicated by periaortitis and cranial hypertrophic pachymeningitis: A report of an autopsy case. *Intern Med*. 2018 Jan 1;57(1):107-113. doi: 10.2169/internalmedicine.8751-16. Epub 2017 Oct 11. PMID: 29021484; PMCID: PMC5799067.
71. Pawlitzki M, Perosa V, Korsen M, Mawrin C, Bartels C, Huchtemann T. Aggressive spinal cord involvement in granulomatosis with polyangiitis. *Int J Rheum Dis*. 2019 Apr;22(4):756-758. doi: 10.1111/1756-185X.13561. Epub 2019 Apr 4. PMID: 30950232.
72. Katada A, Kishibe K, Nozawa H, et al. A case report of Wegener's granulomatosis with hypertrophic pachymeningitis. *Practica Oto-Rhino-Laryngologica*. 2002;95(11):1101-1106. doi:10.5631/jibirin.95.1101
73. Patarata E, Lopez B, Gordon P. Pachymeningitis as the initial presentation of granulomatosis with polyangiitis: a case series of four patients. *Rheumatology*. 2017;56(suppl_2):kex062.028. doi:10.1093/rheumatology/kex062.028
74. Yoshimoto R, Tanaka K, Kawahata T, et al. Unusual manifestations of giant cell arteritis and granulomatosis with polyangiitis. *Immunol Med*. 2019;42(2):94-98. doi:10.1080/25785826.2019.1657377
75. Yasuhara T, Fukuhara T, Nakagawa M, et al. Wegener granulomatosis manifesting as meningitis: case report. *J Neurosurg*. 2002;97(5):1229-1232. doi:10.3171/jns.2002.97.5.1229
76. Yasuda K, Sainouchi M, Goto M, Murase N, Ohtani R, Nakamura M. A case of myeloperoxidase anti-neutrophil cytoplasmic antibody (MPO-ANCA)-associated hypertrophic pachymeningitis presenting with multiple cranial nerve palsies and diabetes insipidus. *Rinsho Shinkeigaku*. 2016;56(5):334-337. doi:10.5692/clinicalneurol.cn-000859
77. Yajima R, Toyoshima Y, Wada Y, et al. A fulminant case of granulomatosis with polyangiitis with meningeal and parenchymal involvement. *Case Rep Neurol*. 2015;7(1):101-104. doi:10.1159/000381942
78. Xiao X, Fu D, Feng L. Hypertrophic pachymeningitis in a southern Chinese population: a retrospective study. *Front Neurol*. 2020;11:565088. doi:10.3389/fneur.2020.565088
79. Watanabe K, Tani Y, Kimura H, et al. Hypertrophic cranial pachymeningitis in MPO-ANCA-related vasculitis: a case report and literature review. *Fukushima J Med Sci*. 2013;59(1):56-62. doi:10.5387/fms.59.56
80. von Bierbrauer A, Pelzer C, Fischer V. Severe chronic headache with depression induced by pachymeningitis in Wegener’s granulomatosis. *Dtsch Med Wochenschr*. 2008;133(5):185-188. doi:10.1055/s-2008-1017494
81. Vasconcelos IMD, Assunção GLMD, Serra JPC, Pereira A, Oliveira EP, Barbas CSV. A generalized refractory granulomatosis with polyangiitis: multiple therapy. In: B40. Autoimmune Lung Disease Case Reports. *Am Thorac Soc Int Conf Abstracts*. American Thoracic Society; 2019:A3088. doi:10.1164/ajrccm-conference.2019.199.1_MeetingAbstracts.A3088
82. Uruha A, Koide R, Bandoh M, Isozaki E, Yoshida H. Neuro-ophthalmic manifestations of hypertrophic cranial pachymeningitis associated with antineutrophil cytoplasmic antibody for myeloperoxidase. *Neuro-Ophthalmol*. 2010;34(4):308-310. doi:10.3109/01658101003778242
83. Yokoseki A, Saji E, Arakawa M, et al. Hypertrophic pachymeningitis: significance of myeloperoxidase anti-neutrophil cytoplasmic antibody. *Brain*. 2014;137(Pt 2):520-536. doi:10.1093/brain/awt314
84. Tsuda R, Taki H, Shinoda K, Hounoki H, Tobe K. Hypertrophic pachymeningitis in an individual with microscopic polyangiitis. *J Am Geriatr Soc*. 2013;61(6):1043-1044. doi:10.1111/jgs.12291
85. Thajeb P, Tsai JJ. Cerebral and oculorhinal manifestations of a limited form of Wegener’s granulomatosis with c-ANCA-associated vasculitis. *J Neuroimaging*. 2001;11(1):59-63. doi:10.1111/j.1552-6569.2001.tb00012.x
86. Tateyama K, Kodama S, Nomi N, Suzuki M, Kishibe K, Harabuchi Y. Serological study of otitis media with ANCA associated vasculitis (OMAAV). *Nihon Jibiinkoka Gakkai Kaiho*. 2015;118(9):1133-1142. doi:10.3950/jibiinkoka.118.1133
87. Takuma H, Shimada H, Inoue Y, et al. Hypertrophic pachymeningitis with anti-neutrophil cytoplasmic antibody (p-ANCA) and diabetes insipidus. *Acta Neurol Scand*. 2001;104(6):397-401. doi:10.1034/j.1600-0404.2001.00056.x
88. Takewaki D, Tsuji Y, Kasai T, Yoshida T, Nakagawa M, Mizuno T. A case of MPO-ANCA positive hypertrophic pachymeningitis associated with vascular inflammation in the kidney biopsy. *Rinsho Shinkeigaku*. 2015;55(11):844-847. doi:10.5692/clinicalneurol.cn-000719
89. Takazawa T, Ikeda K, Nagaoka T, et al. Wegener granulomatosis-associated optic perineuritis. *Orbit*. 2014;33(1):13-16. doi:10.3109/01676830.2013.841716
90. Takahashi Y, Hineno A, Abe R, Yoshida T, Ishii W, Ikeda S. A case of microscopic polyangiitis with hypertrophic pachymeningitis and diabetes insipidus. *Nihon Naika Gakkai Zasshi*. 2015;104(8):1635-1638. doi:10.2169/naika.104.1635
91. Spranger M, Schwab S, Meinck HM, et al. Meningeal involvement in Wegener’s granulomatosis confirmed and monitored by positive circulating antineutrophil cytoplasm in cerebrospinal fluid. *Neurology*. 1997;48(1):263-265. doi:10.1212/wnl.48.1.263
92. Specks U, Moder KG, McDonald TJ. Meningeal involvement in Wegener granulomatosis. *Mayo Clin Proc*. 2000;75(8):856-859. doi:10.4065/75.8.856
93. Soriano A, Lo Vullo M, Casale M, Quattrocchi CC, Afeltra A. Meningeal involvement in Wegener granulomatosis: case report and review of the literature. *Int J Immunopathol Pharmacol*. 2012;25(4):1137-1141. doi:10.1177/039463201202500431
94. Shu J, Gu C, Ren Y, Wei W. ANCA-associated hypertrophic spinal pachymeningitis presenting with longitudinally extensive transverse myelitis: a case report. *Ann Indian Acad Neurol*. 2020;23(1):131-132. doi:10.4103/aian.AIAN_175_19
95. Shimojima Y, Kishida D, Nomura S, Sekijima Y. Cerebrospinal fluid levels of BAFF and APRIL as direct indicators of disease activity in anti-neutrophil cytoplasmic antibody-related hypertrophic pachymeningitis. *Clin Rheumatol*. 2020;39(10):3145-3148. doi:10.1007/s10067-020-05270-6
96. Shimojima Y, Kishida D, Ichikawa T, et al. Hypertrophic pachymeningitis in ANCA-associated vasculitis: a cross-sectional and multi-institutional study in Japan (J-CANVAS). *Arthritis Res Ther*. 2022;24(1):204. doi:10.1186/s13075-022-02898-4
97. Selewski D, Mukherji SK, Kershaw D. A unique neurological presentation of Wegener’s granulomatosis. *Pediatr Nephrol*. 2010;25(8):1567-1568. doi:10.1007/s00467-010-1482-5
98. Sato-Akushichi M, Kinouchi R, Kawai N, Nomura K. Optic neuropathy secondary to granulomatosis with polyangiitis in a patient with Graves’ disease: a case report. *J Med Case Reports*. 2021;15:618. doi:10.1186/s13256-021-03207-4
99. Sakairi T, Sakurai N, Nakasatomi M, et al. Hypertrophic pachymeningitis associated with antineutrophil cytoplasmic antibody-associated vasculitis: a case series of 15 patients. *Scand J Rheumatol*. 2019;48(3):218-224. doi:10.1080/03009742.2018.1498916
100. Saito T, Fujimori J, Yoshida S, Kaneko K, Kodera T. Case of cerebral venous thrombosis caused by MPO-ANCA associated hypertrophic pachymeningitis. *Rinsho Shinkeigaku*. 2014;54(10):827-830. doi:10.5692/clinicalneurol.54.827
101. Saeki T, Fujita N, Kourakata H, Yamazaki H, Miyamura S. Two cases of hypertrophic pachymeningitis associated with myeloperoxidase antineutrophil cytoplasmic autoantibody (MPO-ANCA)-positive pulmonary silicosis in tunnel workers. *Clin Rheumatol*. 2004;23(1):76-80. doi:10.1007/s10067-003-0815-1
102. Peng W, Wang X. Hypertrophic pachymeningitis and cerebral infarction resulting from ANCA-associated vasculitis. *Neurol India*. 2012;60(4):424-426. doi:10.4103/0028-3886.100711
103. Park JH. ANCA-associated hypertrophic pachymeningitis and optic neuropathy: report of 2 new cases and review of the literature. *J Neurol Sci*. 2021;429:118485. doi:10.1016/j.jns.2021.118485
104. Ohashi K, Morishita M, Watanabe H, et al. Central diabetes insipidus in refractory antineutrophil cytoplasmic antibody-associated vasculitis. *Intern Med*. 2017;56(21):2943-2948. doi:10.2169/internalmedicine.8683-16
105. Ogaki R, Okada E, Suzuki S, et al. Myeloperoxidase-antineutrophil cytoplasmic antibody positive hypertrophic spinal pachymeningitis at the cervicothoracic junction: a case report. *Spine Surg Relat Res*. 2021;5(3):211-213. doi:10.22603/ssrr.2020-0066
106. Nishiike S, Kato T, Nagai M, Konishi M, Sakata Y. Management and follow-up of localized Wegener’s granulomatosis: a review of five cases. *Acta Otolaryngol*. 2004;124(9):1103-1108. doi:10.1080/00016480410020310
107. Nguyen T, Vandergheynst F. Pachymeningitis and aortitis in a patient with antineutrophil cytoplasmic antibody-associated vasculitis. *Mayo Clin Proc*. 2014;89(11):e115. doi:10.1016/j.mayocp.2014.03.021
108. Nakajima A. Characterization of spinal hypertrophic pachymeningitis based on immunopathological analysis. Presented at: *Neuro2021*. Accessed April 16, 2025. <https://confit.atlas.jp/guide/event/neuro2021/subject/Pe-09-1/detail>
109. Nagaoka T, Ikeda K, Hirayama T, Yamamoto T, Iwasaki Y. Wegener granulomatosis-associated optic perineural hypertrophy and optic neuropathy. *Intern Med*. 2012;51(2):227-228. doi:10.2169/internalmedicine.51.6532
110. Morán-Castaño C, Suárez-Díaz S, Álvarez-Marcos CA, et al. ANCA-associated hypertrophic pachymeningitis, a central nervous system limited type of systemic vasculitis. *QJM*. 2023;116(3):241-243. doi:10.1093/qjmed/hcac235
111. Mentzel HJ, Neumann T, Fitzek C, Sauner D, Reichenbach JR, Kaiser WA. MR imaging in Wegener granulomatosis of the spinal cord. *AJNR Am J Neuroradiol*. 2003;24(1):18-21.
112. McCarthy PJ, Arend WP, Kleinschmidt-DeMasters BK. May 2001: 32 year old female with dural mass encircling cervical spinal cord. *Brain Pathol*. 2001;11(4):483-484, 487. doi:10.1111/j.1750-3639.2001.tb01090.x
113. Matsumoto K, Akiyama M, Kajio N, et al. Adolescent PR3-ANCA-positive hypertrophic pachymeningitis. *Medicine (Baltimore)*. 2018;97(17):e0521. doi:10.1097/MD.0000000000010521
114. López-Rodríguez R, García-González J, Campos-Franco J, Mallo-González N, Alende-Sixto MR. Neurological involvement in Wegener’s granulomatosis: report of one case. *Rev Med Chil*. 2007;135(7):913-916. doi:10.4067/s0034-98872007000700012
115. Li X, Zhao J, Wang Q, Fei Y, Zhao Y. ANCA-associated systemic vasculitis presenting with hypertrophic spinal pachymeningitis: a report of 2 cases and review of literature. *Medicine (Baltimore)*. 2015;94(46):e2053. doi:10.1097/MD.0000000000002053
116. Li S, Tang H, Rong X, Huang X, Li Q. Pachymeningitis as a manifestation of ANCA-associated vasculitis: a case report and literature review. *Int J Clin Exp Med*. 2015;8(4):6352-6359.
117. Kurihara Y, Oku K, Suzuki A, et al. A case of slowly progressive type 1 diabetes mellitus developing myeloperoxidase-specific anti-neutrophil cytoplasmic antibody-associated vasculitis with hypertrophic pachymeningitis manifesting as multiple cranial nerve palsy. *Nihon Rinsho Meneki Gakkai Kaishi*. 2011;34(6):510-515. doi:10.2177/jsci.34.510
118. Kumamoto M, Tomoda K, Furuya Y, et al. Hypertrophic pachymeningitis as a delayed complication of granulomatosis with polyangiitis. *Intern Med*. 2016;55(4):413-417. doi:10.2169/internalmedicine.55.5434
119. Kuhn J, Weber M, Hedde JP, Bewermeyer H. Atypical headache and facial pain as a result of hypertrophic pachymeningitis in C-ANCA-positive Wegener’s granulomatosis. *Med Klin (Munich)*. 2005;100(4):209-212. doi:10.1007/s00063-005-1023-3
120. Koura T, Kita K, Konishi H. A case of suspected MPO-ANCA-related hypertrophic pachymeningitis with atypical presentation. *J Gen Fam Med*. 2017;18(6):409-410. doi:10.1002/jgf2.73
121. Koide S, Kitajima K, Yamazaki M, Ichikawa T, Komatsu M. Hypertrophic pachymeningitis associated with myeloperoxidase-anti-neutrophil cytoplasmic antibodies induced by propylthiouracil. *Intern Med*. 2023;62(13):1951-1955. doi:10.2169/internalmedicine.0692-22
122. Kohlberg GD, Truong MT, Chang KW. Wegener’s granulomatosis in an adolescent presenting with pachymeningitis, mastoid effusion and Horner’s syndrome. *Int J Pediatr Otorhinolaryngol Extra*. 2011;6(2):80-84. doi:10.1016/j.pedex.2010.03.009
123. Kiyohara M, Shirai T, Nishiyama S, et al. Hypertrophic pachymeningitis development in eosinophilic granulomatosis with polyangiitis at relapse of disease: a case-based review. *Tohoku J Exp Med*. 2022;256(3):241-247. doi:10.1620/tjem.256.241
124. Kira YI, Yoshikawa Y, Tashiro T, Maeda N. Granulomatosis with polyangiitis presenting with thrombovasculitic and necrotizing pachy- and leptomeningitis accompanied by a brain tumor-like lesion. *Intern Med*. 2023;62(21):3241-3246. doi:10.2169/internalmedicine.0833-22
125. Kazmi M, Akil M, Kilding R. Granulomatosis with polyangiitis presenting with multiple cranial nerve palsies. *BMC Musculoskelet Disord*. 2013;14(Suppl 1):A11. doi:10.1186/1471-2474-14-S1-A11
126. Kamimura T, Shimazaki H, Morita M, Nakano I, Okazaki H, Minota S. Limited Wegener’s granulomatosis manifested by abducens nerve palsy resulting from pachymeningitis. *J Clin Rheumatol*. 2006;12(5):259-260. doi:10.1097/01.rhu.0000239904.62352.5e
127. Kaieda S, Yoshida N, Minezaki M, et al. The successful treatment of myeloperoxidase antineutrophil cytoplasmic antibody-positive hypertrophic pachymeningitis in patients with the limited form of granulomatosis with polyangiitis using methotrexate: two case reports. *Intern Med*. 2017;56(8):959-965. doi:10.2169/internalmedicine.56.7742
128. Just SA, Knudsen JB, Nielsen MK, Junker P. Wegener’s granulomatosis presenting with pachymeningitis: clinical and imaging remission by rituximab. *ISRN Rheumatol*. 2011;2011:608942. doi:10.5402/2011/608942
129. Jinnah HA, Dixon A, Brat DJ, Hellmann DB. Chronic meningitis with cranial neuropathies in Wegener’s granulomatosis: case report and review of the literature. *Arthritis Rheum*. 1997;40(3):573-577. doi:10.1002/art.1780400326
130. Jacobi D, Maillot F, Hommet C, et al. P-ANCA cranial pachymeningitis: a case report. *Clin Rheumatol*. 2005;24(2):174-177. doi:10.1007/s10067-004-1022-4
131. Iqbal AM, Blackburn D, Rafiq M, Sharrack B. Wegener’s granulomatosis presenting with multiple cranial nerve palsies and pachymeningitis. *Pract Neurol*. 2013;13(3):193-195. doi:10.1136/practneurol-2012-000340
132. Imafuku A, Sawa N, Kawada M, et al. Incidence and risk factors of new-onset hypertrophic pachymeningitis in patients with anti-neutrophil antibody-associated vasculitis: using logistic regression and classification tree analysis. *Clin Rheumatol*. 2019;38(4):1039-1046. doi:10.1007/s10067-018-4372-z
133. Ikeda K, Takazawa T, Nagaoka T, et al. Hypertrophic perioptic neuritis (HPN) in Wegener granulomatosis (WG). In: Abstracts of the Meeting. 2012:S29-S30.
134. Igarashi S, Otani T, Takahashi YK, Soga K, Irioka T, Yokota T. Clinical features of two cases of deafness related to antineutrophil cytoplasmic antibodies-associated vasculitis. *J Neurol Sci*. 2017;381:541. doi:10.1016/j.jns.2017.08.3731
135. Huang YH, Ro LS, Lyu RK, et al. Wegener’s granulomatosis with nervous system involvement: a hospital-based study. *Eur Neurol*. 2015;73(3-4):197-204. doi:10.1159/000375492
136. Hota D. Pachymeningitis as a presenting manifestation of Wegener’s granulomatosis. *J Neurol Sci*. 2019;405:24. doi:10.1016/j.jns.2019.10.807
137. Hoshino R, Furuta M, Shibata M, et al. Vanishing tumefactive ANCA-associated hypertrophic pachymeningitis: a case report. *Clin Neurol Neurosurg*. 2022;212:107070. doi:10.1016/j.clineuro.2021.107070
138. Horino T, Takao T, Taniguchi Y, Terada Y. Hypertrophic pachymeningitis with MPO-ANCA-positive vasculitis. *Clin Rheumatol*. 2010;29(1):111-113. doi:10.1007/s10067-009-1269-x
139. Hayashi S, Sugeno N, Nishiyama S, Hasegawa T, Aoki M. Unusual visual impairments in a case of MPO-ANCA associated hypertrophic pachymeningitis. *Rinsho Shinkeigaku*. 2012;52(3):152-155. doi:10.5692/clinicalneurol.52.152
140. Hayashi K, Watanabe H, Yamamura Y, et al. Granulomatosis with polyangiitis with obstructive pneumonia progressing to hypertrophic pachymeningitis: a case report. *Medicine (Baltimore)*. 2021;100(3):e24028. doi:10.1097/MD.0000000000024028
141. Hahn LD, Fulbright R, Baehring JM. Hypertrophic pachymeningitis. *J Neurol Sci*. 2016;367:278-283. doi:10.1016/j.jns.2016.06.024
142. Gupta V, Sharma AK, Sureka RK, Bhuyan SK, Singh PK. Chronic meningitis with multiple cranial neuropathies: a rare initial presentation of Wegener’s granulomatosis. *Ann Indian Acad Neurol.* 2013;16(3):411-413. doi:10.4103/0972-2327.116920
143. Gu Y, Sun X, Peng M, Zhang T, Shi J, Mao J. Pituitary involvement in patients with granulomatosis with polyangiitis: case series and literature review. *Rheumatol Int.* 2019;39(8):1467-1476. doi:10.1007/s00296-019-04338-0
144. Gallo C, Galli G, Strigaro G, et al. Pachymeningitis in granulomatosis with polyangiitis: a case report. *J Neurol Sci.*2021;429:118791. doi:10.1016/j.jns.2021.118791
145. Furuya Y, Suzuki M, Abe H, et al. Wegener’s granulomatosis with pachymeningitis. *Nihon Naika Gakkai Zasshi.*2002;91(12):3510-3512. doi:10.2169/naika.91.3510
146. Funauchi M, Yoo BS, Sugiyama M, et al. A case of rheumatoid meningitis positive for perinuclear antineutrophil cytoplasmic antibody. *Ann Rheum Dis.* 2000;59(12):1001-1002. doi:10.1136/ard.59.12.1001a
147. Fam AG, Lavine E, Lee L, Perez-Ordonez B, Goyal M. Cranial pachymeningitis: an unusual manifestation of Wegener’s granulomatosis. *J Rheumatol.* 2003;30(9):2070-2074.
148. Escobar MCM, Neeley B, Ebbert M, Shahabadi AZ, Akter S, Sriwastava S. Case series of hypertrophic pachymeningitis with underlying etiology (122). *Neurology.* 2021;96(15_suppl):122. doi:10.1212/WNL.96.15_supplement.122
149. El Aoud S, Frikha F, Ben Salah R, Snoussi M, Loukil H, Bahloul Z. Multiple cranial nerve palsy revealing hypertrophic pachymeningitis with positive myeloperoxidase-antineutrophil cytoplasmic antibody. *Reumatismo.*2013;65(5):248-252. doi:10.4081/reumatismo.2013.248
150. Duarte AC, Sousa S, Cordeiro A. AB0582 Neurologic involvement in antineutrophil cytoplasmic antibody (ANCA)-associated vasculitis – experience from a Portuguese center. *Ann Rheum Dis.* 2019;78:1753. doi:10.1136/annrheumdis-2019-eular.481
151. Dörr J, Elitok S, Dieste FJ, et al. Treatment-resistant chronic headaches and focal pachymeningitis in a 46-year-old man: a rare presentation of Wegener’s granulomatosis. *Lancet Neurol.* 2008;7(4):368-372. doi:10.1016/S1474-4422(08)70064-4
152. De Luna G, Terrier B, Charles P, et al. AB0453 Presentation and management of granulomatosis with polyangiitis (Wegener’s) (GPA) central nervous system (CNS) involvement. *Ann Rheum Dis.* 2013;72:A927. doi:10.1136/annrheumdis-2013-eular.2775
153. de Hoog J, Volovici V, Dammers R. Successful surgical optic nerve decompression in a patient with hypertrophic pachymeningitis due to granulomatous polyangiitis. *BMJ Case Rep.* 2015;2015:bcr2014208110. doi:10.1136/bcr-2014-208110
154. Cossack M, Vanam S, Wallace B. Vision loss with pachymeningeal enhancement on magnetic resonance imaging. *JAMA Ophthalmol.* 2017;135(5):495-496. doi:10.1001/jamaophthalmol.2016.4542
155. Clément M, Néel A, Toulgoat F, et al. Inflammatory optic neuropathy in granulomatosis with polyangiitis can mimic isolated idiopathic optic neuritis. *Eur J Ophthalmol.* 2021;31(1):245-251. doi:10.1177/1120672119889008
156. Choi YJ, Choi JY, Yang HK. Orbital apex syndrome with perinuclear anti-neutrophil cytoplasmic antibody-associated pachymeningitis. *J Korean Ophthalmol Soc.* 2020;61(5):587-590.
157. Choi JR, Park W. A case of cortical vein thrombosis in Wegener’s granulomatosis. *J Rheum Dis.* 2012;19(2):108-111. doi:10.4078/jrd.2012.19.2.108
158. Cho J, Carabenciov I, Wieland M. MPO-ANCA vasculitis pachymeningitis with bilateral vision loss. *Minn Med.*2015;98(8):41.
159. Chen H, Zhang W, Jing J, et al. The clinical and imaging features of hypertrophic pachymeningitis: a clinical analysis on 22 patients. *Neurol Sci.* 2019;40(2):269-274. doi:10.1007/s10072-018-3619-4
160. Caramaschi P, Biasi D, Carletto A, Bambara LM. Vascularite à ANCA avec atteinte prédominante du système nerveux central: une observation. *Rev Rhum.* 2003;70(9):762-765. doi:10.1016/S1169-8330(03)00266-7
161. Cannon PS, Cruz AAV, Pinto CT, et al. A multi-centre case series investigating the aetiology of hypertrophic pachymeningitis with orbital inflammation. *Orbit.* 2011;30(2):64-69. doi:10.3109/01676830.2010.539766
162. Burrell HC, McConachie NS. Pachymeningitis in Wegener’s granulomatosis. *Australas Radiol.* 1998;42(4):364-366. doi:10.1111/j.1440-1673.1998.tb00539.x
163. Blech B, Meyer M, Goodman B, et al. Vasculitis presenting as autonomic failure. *The Neurologist.* 2021;26(4):117-121. doi:10.1097/NRL.0000000000000327
164. Bi Z, Shang K, Cao J, et al. Hypertrophic pachymeningitis in Chinese patients: presentation, radiological findings, and clinical course. *Biomed Res Int.* 2020;2020:2926419. doi:10.1155/2020/2926419
165. Barbieri FR, Novegno F, Iaquinandi A, Lunardi P. Hypertrophic pachymeningitis and hydrocephalus—the role of neuroendoscopy: case report and review of the literature. *World Neurosurg.* 2018;119:183-188. doi:10.1016/j.wneu.2018.07.194
166. Bahrami B, Juniat V, Davis G, Selva D. Pachymeningeal enhancement on magnetic resonance imaging in granulomatosis with polyangiitis. *Can J Ophthalmol.* 2020;55(5):e181-e182. doi:10.1016/j.jcjo.2020.04.005
167. Astouati Q, Provot F, Farhat MM, Launay D, Sanges S. Use of avacopan in severe forms of ANCA-associated vasculitis. Comment on “Inhibiting C5a/C5aR to treat ANCA-associated vasculitides” by Terrier et al. *Joint Bone Spine.* 2023;90(2):105522. doi:10.1016/j.jbspin.2023.105522
168. Arnaoutoglou MA, Xerras CG, Kalevrosoglou IK, Rafailidis VD, Notas KP, Tegos TI. Headache linked to intracranial hypertension and hypertrophic pachymeningitis as the initial and dominant presentation of granulomatosis with polyangiitis. Case report and review of the recent literature. *Headache.* 2018;58(4):589-595. doi:10.1111/head.13277
169. Antunes AP, Pimentel J. Hypertrophic pachymeningitis: a continuum process? *F1000Research.* 2011;2. Accessed April 16, 2025. <https://f1000research.com/posters/1806>
170. Abe T, Nogawa S, Tanahashi N, Shiraishi J, Ikeda E, Suzuki N. Cerebral pachyleptomeningitis associated with MPO-ANCA induced by PTU therapy. *Intern Med.* 2007;46(5):247-250. doi:10.2169/internalmedicine.46.1859
171. Takahashi K, Kobayashi S, Okada K, Yamaguchi S. Pachymeningitis with a perinuclear antineutrophil cytoplasmic antibody: response to pulse steroid. *Neurology.* 1998;50(4):1190-1191. doi:10.1212/WNL.50.4.1190
172. Costa C, Santiago T, Espirito-Santo J, Rovisco J, Silva J, Malcata A. Pachymeningitis and cerebral granuloma in granulomatosis with polyangiitis: is rituximab a promising treatment option? *Acta Reumatol Port.* 2017;42(1):82-87. PMID:28371573.
173. Ito K, Umemura K, Suzuki A, et al. Microscopic polyangiitis (MPA) accompanied with hypertrophic pachymeningitis (HP). *The Japanese Society of Neuropathology, Abstracts of the 53rd Annual Meeting*; June 28–30, 2012; Niigata, Japan.
174. Wang Y, Wang H, Jia L, Akwilini A, Fu R. Severe headache with fever: intracranial infections or relapses of ANCA-associated vasculitis? Case report with literature review. *Nephrology*. 2020;25(Suppl 1):22-95.
175. ​​Perez C, Gonzalez M, Fanlo P, Huarte E, Lacruz B, Garcia-Bragado F. Successful treatment with RTX in a refractory WG with leucoencephalopathy and pachymeningitis. *Clin Exp Immunol*. 2011;164:103-112. doi:10.1111/j.1365-2249.2011.04343.x
176. Carmen P, Dumitrescu L, Caraiola S, et al. Thoracolumbar pachymeningitis causing bilateral subacute radicular compression in Wegener’s granulomatosis: A case report. *Eur J Neurol*. 2017;24:123-444. doi:10.1111/ene.13367
177. Flores-Suárez LF, Rodríguez M. Unilateral multiple cranial nerve palsy in Wegener's granulomatosis. *Clin Exp Immunol*. 2011;164:103-112. doi:10.1111/j.1365-2249.2011.04343.x

**eFigure 1**. Flow diagram


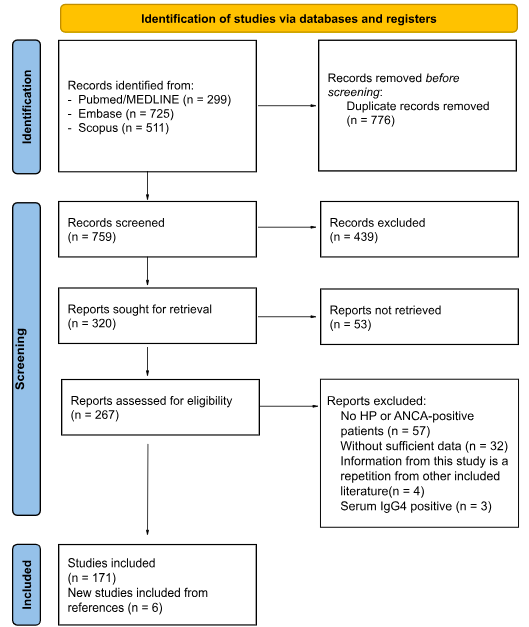

HP: Hypertrophic pachymeningitis; ANCA: Antineutrophil cytoplasmatic antibody; IgG4: Immunoglobulin G4
